# Supplementary material for: A whole-animal phenotypic drug screen identifies suppressors of atherogenic lipoproteins
Source: eLife. 2026 Aug 27;14:RP105314. doi: 10.7554/eLife.105314 (PMC13521566; doi:10.7554/eLife.105314)
Supplement: Supplementary file 2 [file elife-105314-code1.zip › JHDL_validation_result/Danazol_JB20200217_01.pdf]

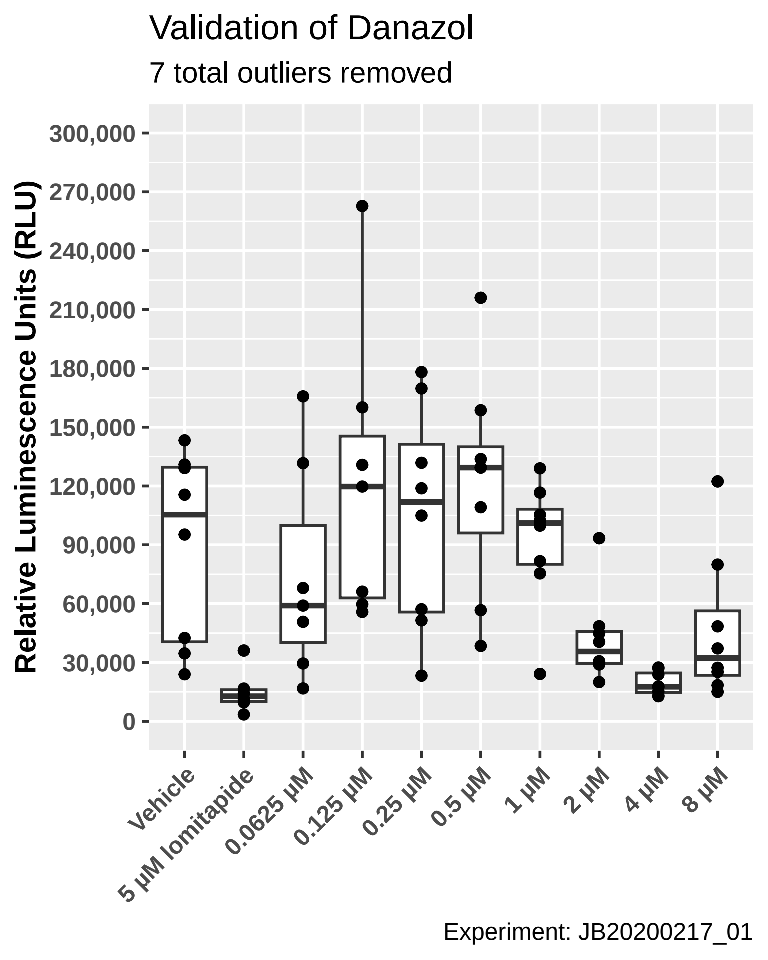

Validation of Danazol
7 total outliers removed
Relative Luminescence Units (RLU)
300,000
270,000
240,000
210,000
180,000
150,000
120,000
90,000
60,000
30,000
0
Vehicle
5 μM lomitapide
0.0625 μM
0.125 μM
0.25 μM
0.5 μM
1 μM
2 μM
4 μM
8 μM
Experiment: JB20200217_01
